# Rapid Preparation of Carrier-Free Self-Assembled Aqueous Nanoformulations of Ten BCS Class II Drugs, Propofol, and Hydroxyapatite Using FAST Nanotechnology

**DOI:** 10.26502/fjhs.434

**Published:** 2026-08-12

**Authors:** Stephen Hsu, Hongfang Yu, Archith Philip, Amani Mouna, Brendan Marshall, Douglas Dickinson

**Affiliations:** 1 Department of Oral Biology, Dental College of Georgia, Augusta University, Augusta, GA. USA; 2 Department of Cellular Biology and Anatomy, Medical College of Georgia, Augusta University, Augusta, GA. USA; 3 College of Science and Mathematics, Augusta University, Augusta, GA. USA.; 4 Electron Microscopy and Histology Core lab, Augusta University, Augusta, GA. USA.; 5 Camellix Research Laboratory, Life Sciences Business Development Center, Augusta University, Augusta, GA. USA.

**Keywords:** Nanoparticles, nanoformulations, BCS Class II drugs, Hydroxyapatite, Propofol, Facilitated Self-assembling Technology (FAST)

## Abstract

Poor aqueous solubility remains a major barrier to the development, repurposing, and improved delivery of clinically important small-molecule drugs. This formulation problem is especially relevant to Biopharmaceutics Classification System (BCS) Class II drugs, for which dissolution rather than membrane permeability may limit absorption and translational testing. Here, we evaluated Facilitated Self-Assembling Technology (FAST) as a rapid, carrier-free aqueous nanoformulation platform for ten representative BCS Class II drugs, propofol, and Omyadent HA100 hydroxyapatite as an inorganic oral-care material. FAST generated nanoscale aqueous suspensions across the drug panel, with negative zeta potentials and no visible macroscopic precipitation, sedimentation, aggregation, phase separation, or foam formation under the visual assessment conditions. Transmission electron microscopy of propofol, itraconazole, rifampin, and HA100 suspensions supported diverse nanoscale morphologies, including rounded particles, heterogeneous particle clusters, and irregular mineral clusters. Itraconazole showed coexisting rod-like electron-dense structures consistent with possible crystalline/nanocrystalline material and rounded nanoscale particles, supporting future investigation of morphology-controlled reformulation. FAST-processed HA100 showed nanoscale mineral clusters, indicating that FAST may extend beyond hydrophobic organic drugs to inorganic materials. This study establishes a formulation-feasibility framework and supports further evaluation of FAST as a clean, rapid, carrier-free platform for early formulation screening, value-added reformulation of approved drugs, AI-assisted drug discovery, topical/local delivery concepts, and future oral-care applications, subject to determination of improved dissolution, pharmacokinetics, bioavailability, or efficacy.

## Introduction

Poor aqueous solubility is one of the most common formulation barriers encountered in small-molecule drug development. Many drug candidates and approved drugs have high lipophilicity and limited water compatibility, creating challenges in dissolution, oral absorption, parenteral formulation, topical delivery, biological testing, and dose optimization [1–3]. The Biopharmaceutics Classification System (BCS) provides a useful framework for understanding oral drug absorption. BCS Class II drugs are characterized by low solubility and high permeability; therefore, their absorption may be limited primarily by dissolution rather than membrane transport [2,3].

A wide range of formulation strategies has been developed to address poor solubility, including particle-size reduction, wet milling, high-pressure homogenization, nanocrystals, nanosuspensions, amorphous solid dispersions, lipid-based formulations, self-emulsifying systems, cyclodextrin complexes, cocrystals, and polymeric or surfactant-stabilized systems [1,4]. These technologies have provided important translational value, but they often require drug-specific excipient selection, stabilizers, carrier matrices, high-energy processing, solvent removal, scale-up optimization, and extensive stability testing. A rapid, simple, aqueous, clean, and carrier-free approach could therefore be useful for converting poorly soluble drugs into testable nanoscale dispersions early in development.

Facilitated Self-Assembling Technology (FAST) was recently developed as a practical approach to prepare aqueous nanoparticles from hydrophobic compounds. Previous FAST studies generated nanoparticles from several poorly soluble molecules, and ZetaView/nanoparticle tracking analysis, zeta potential measurement, and transmission electron microscopy were used to evaluate particle size distribution, particle concentration, surface charge, and selected morphology [5–7]. The present study extends this platform to a focused panel of clinically relevant poorly water-soluble drugs and one inorganic oral-care material.

The drug panel was selected to cover formulation-relevant diversity rather than a single therapeutic class. Ten compounds were selected as representative BCS Class II drugs across anti-inflammatory, antifungal, antibiotic, anticancer, cardiovascular, lipid-lowering, neuropsychiatric, anticoagulant, and dermatologic applications. Propofol was included as an additional clinically important hydrophobic drug whose approved product depends on a lipid emulsion, providing a stress-test example for value-added reformulation. Lastly, Omyadent HA100 hydroxyapatite was included as an inorganic dental proof-of-concept material to determine whether FAST processing could yield a nanoparticle-enriched mineral suspension under mild aqueous conditions. Table 1 summarizes the rationale for each material.

The selected drugs vary in size, aromaticity, heteroatom content, hydrogen-bonding capacity, lipophilicity, ionizable groups, solid-state behavior, and therapeutic class [27–37]. This diversity allows FAST to be evaluated as a platform approach rather than as a single-compound formulation method. The structural diversity of the small-molecule panel also provides a rationale for evaluating whether a common carrier-free self-assembly workflow can generate aqueous nanoscale dispersions from chemically distinct poorly water-soluble drugs.

## Materials and Methods

### Compounds and Reagents

Celecoxib (A10193–100), sorafenib (A17857–500), carvedilol (A11843–100), ezetimibe (A10379–50), lurasidone (A11214–50), rivaroxaban (A10800–50), fenofibrate (SKU A10385–5000), and propofol (A18293–100) were purchased from AdooQ BioSciences (Irvine, CA, USA). Itraconazole (J66390.03), rifampin (455620010), and spironolactone (207460010) were obtained from Fisher Scientific (Pittsburgh, PA, USA). Omyadent HA100 hydroxyapatite (Lot# OMD624231B) was provided by Nutrivene/International Nutrition Inc. (Middle River, MD, USA). Sterile double-distilled water, purified 0.1 μm-filtered water, glycerol, paraformaldehyde, glutaraldehyde, and uranyl acetate were used as described below.

### Preparation of Drug Nanoparticles

Drug nanoparticles were prepared using the proprietary Facilitated Self-Assembling Technology (FAST) method, as previously described with modifications [5–7]. The FAST method is patent pending under U.S. Patent Application No. 63/896,918. Briefly, individual poorly water-soluble drugs were processed under compound-specific FAST conditions to generate nanoparticle stock suspensions. The resulting nanoparticle stocks were prepared at 0.1–1% w/v and stabilized in pure glycerol for storage. Before particle characterization and visual suspension evaluation, the glycerol stock suspensions were diluted 50-fold with sterile double-distilled water to nominal working suspension levels, unless otherwise specified. For TEM imaging, the glycerol suspension stocks were diluted 1:4 in purified double-distilled water.

### Nanoparticle Tracking Analysis and Zeta Potential Measurement

Particle size distribution and particle concentration were measured using a ZetaView X20 Nanoparticle Tracking Analyzer (Particle Metrix, Meerbusch, Germany), following a method described previously [5–7]. The instrument measuring range for particle diameter was 10–2000 nm. Samples were diluted in purified 0.1 μm-filtered water before analysis. Measurements were performed at 25°C with sensitivity 70, frame rate 30 frames per second, and shutter speed 100. Particle information was collected from 11 positions across the cell with two reading cycles. Post-acquisition parameters were set as follows: minimum brightness 20, minimum area 10, maximum area 1000, and trace length 15.

For itraconazole, a 0.22 μm filtration step was used before ZetaView/NTA analysis to remove larger rod-like electron-dense structures and enrich the filter-passing nanosuspension fraction. Therefore, the reported itraconazole particle size, particle concentration, and zeta potential values represent the filter-passing fraction rather than the total unfiltered preparation.

Zeta potential was measured using the same ZetaView X20 system. Nanoparticle suspensions were diluted in purified 0.1 μm-filtered water with low conductivity (<1000 μS/cm) before analysis. Zeta potential profiles were collected from 11-position measurements at 25 °C. Particle size, particle concentration, and zeta potential values were recorded for each nanoparticle suspension.

### Transmission Electron Microscopy

Transmission electron microscopy was used to examine the morphology of selected drug nanoparticles. Drug nanoparticle stocks were diluted with double-distilled water and fixed in 4% paraformaldehyde and 2% glutaraldehyde. After mixing, 5 μL of each sample was transferred to a Formvar/carbon-coated copper 200-mesh grid and allowed to dry for 15 minutes. Excess liquid was removed using filter paper. Samples were then negatively stained by adding 5 μL of 2% aqueous uranyl acetate. Multiple images were captured from each sample using a JEM 1400 Flash transmission electron microscope (JEOL, Peabody, MA, USA) operated at 120 kV with a Gatan OneView digital camera (Gatan Inc., Pleasanton, CA, USA).

### Visual Assessment of Drug Nanoparticle Water Suspensions

Drug nanoparticle stocks were diluted in double-distilled water to nominal working suspension levels of 0.01–0.02% to generate aqueous nanoparticle suspensions. These percentage values are preparation/dilution designations and were not analytically confirmed as w/v or w/w drug concentrations.

To evaluate short-term visual dispersion stability, diluted nanoparticle suspensions were stored at room temperature for at least 10 days. After storage, the aged diluted suspensions were photographed in glass tubes alongside freshly diluted nanoparticle suspensions of the same drugs. Visual assessment of color change, visible precipitation, sedimentation, and aggregation was performed using coded samples by three independent observers before documentation was finalized.

## Results

### ZetaView/NTA Characterization of FAST-Generated Aqueous Nanoscale Suspensions

FAST processing generated measurable aqueous nanoscale suspensions across the selected poorly water-soluble drug panel, propofol, and Omyadent HA100. Measured particle-size distribution profiles are shown in Figure 2, and the corresponding quantitative data are compiled in Table 2.

### Visual Appearance and Short-Term Suspension Sta-bility

Diluted FAST-generated aqueous suspensions were visually compared as pairs of aged samples (stored for at least 10 days at room temperature) and freshly prepared samples. The paired visual assessment was organized into two panels to allow direct comparison of each suspension. Group 1 included carvedilol, celecoxib, ezetimibe, fenofibrate, itraconazole, and lurasidone. Group 2 included propofol, rifampin, rivaroxaban, sorafenib, spironolactone, and HA100.

No macroscopic precipitation, sedimentation, aggregation, phase separation, or foam formation was observed by visual inspection for the evaluated suspensions. Most aged and freshly prepared pairs remained visually similar in color and overall appearance. Rifampin was the only sample that showed a clear visual color shift, with the aged suspension appearing darker yellow/amber compared with the freshly prepared rifampin suspension.

### TEM evaluation of selected representative FAST-generated nanoparticles

TEM imaging was used to examine selected representative FAST-generated nanoparticle suspensions. Propofol was selected as a clinically important hydrophobic small molecule whose marketed formulation depends on an oil-in-water lipid emulsion. Itraconazole was selected as a highly lipophilic azole antifungal drug with potential topical, ocular, and local antifungal formulation relevance. Rifampin was selected because of its amphipathic macrocyclic structure, aqueous formulation sensitivity, and relevance to nanoparticle-based tuberculosis delivery concepts. Omyadent HA100 was evaluated by TEM as an inorganic mineral proof-of-concept material.

FAST-generated propofol nanoparticles appeared as abundant discrete spherical to near-spherical particles distributed across the TEM field. The observed particles were in the nanoscale range and were broadly consistent with the ZetaView/NTA particle-size results. The field showed some size heterogeneity, suggesting a polydisperse nanoparticle population, but no obvious needle-like or crystalline morphology was observed.

FAST-generated itraconazole showed two distinct nanoscale morphologies by TEM. One field showed rod-like electron-dense structures consistent with possible crystalline/nanocrystalline material, while a separate field showed rounded nanoscale particles with darker internal substructure. These images demonstrate coexisting morphologies within the FAST preparation. Because the 0.22 μm filtration step removed larger structures before ZetaView/NTA analysis, future fraction-specific characterization is required to establish composition, solid state, recovery, and release behavior before morphology-controlled reformulation is proposed.

FAST-generated rifampin showed heterogeneous nanoscale morphology by TEM. Representative fields showed irregular clustered aggregates composed of smaller nanoscale substructures and separate rounded to oval nanoscale particles within a diffuse matrix. No long needle-like crystalline morphology was observed. Because the corresponding aqueous rifampin suspension appeared clear brown/amber without visible macroscopic precipitation or sedimentation, the aggregated TEM structures are interpreted cautiously as nanoscale rifampin particle clusters and/or drying/staining-associated aggregates rather than direct evidence of bulk precipitation.

FAST-processed Omyadent HA100 showed abundant electron-dense, irregular, granular nanoscale clusters distributed across the TEM field. The morphology was not uniform or spherical; instead, the HA appeared as irregular nanoscale clusters composed of finer electron-dense substructures. Some larger aggregates were observed, indicating that the preparation represents a nanoparticle-enriched HA suspension rather than complete conversion into monodisperse isolated nanoparticles.

## Discussion

### Overall interpretation of FAST-generated aqueous nanoscale suspensions

The broader formulation literature supports the rationale for nanoscale approaches in poorly soluble drug development. Nanocrystal and nanosuspension reviews describe increased surface area, improved dissolution, improved apparent solubility, and opportunities for integration into tablets, hydrogels, microneedles, microparticles, and functionalized systems as key advantages for poorly soluble drugs [4,38,39]. The present study evaluated FAST as a rapid formulation-feasibility platform for converting chemically diverse poorly water-soluble molecules into aqueous nanoscale suspensions. FAST differs from many conventional approaches because the current method is intended to be rapid, aqueous, carrier-free, and low-energy, although direct head-to-head comparisons with established nanocrystal, solid dispersion, lipid, and surfactant-based systems remain necessary.

Across the drug panel, ZetaView/NTA identified measurable nanoscale particle populations, with nanoscale morphology supported in selected cases by TEM. Zeta potential measurements showed negative surface charge for all tested suspensions. The visual stability assessment further showed no macroscopic precipitation, sedimentation, aggregation, phase separation, or foam formation under the evaluated conditions, although rifampin displayed a visible yellow-to-darker-yellow/amber color shift after aging. This observation is consistent with the known aqueous color/stability sensitivity of rifampin and should be confirmed by analytical stability methods such as HPLC or LC-MS. These findings support the central conclusion that FAST can rapidly generate aqueous nanoparticle-enriched suspensions of diverse compounds suitable for further formulation development. The present study provides the basis for future testing to determine whether dissolution, pharmacokinetics, bioavailability, or efficacy are improved.

### Value-added reformulation opportunities for BCS Class II drugs and related hydrophobic drugs

The ten BCS Class II drugs and propofol were selected to represent formulation-relevant diversity, rather than exhaustive coverage of one therapeutic area. The overall rationale is summarized in Table 1, while more detailed drug-specific reformulation opportunities are considered below. The phrase “value-added reformulation” is used to describe improved formulations of known or approved active ingredients that may address limitations in dissolution, dispersion uniformity, dose flexibility, route of administration, local retention, tolerability, or product design. The approved adult dosing requirements for these compounds span a wide range. Celecoxib is commonly administered at 200 mg/day, with approved regimens of up to 200 mg twice daily depending on indication [43]. Fenofibrate dosing is formulation dependent and is commonly approximately 48–160 mg once daily [44]. Itraconazole capsules are generally administered at 200 mg/day, which may be increased to 400 mg/day for selected systemic infections [45]. For tuberculosis, adult rifampin is administered at 10 mg/kg once daily, not to exceed 600 mg/day [46]. Sorafenib is administered at 400 mg twice daily (800 mg/day) [47], whereas spironolactone dosing ranges from 25 mg/day for heart failure to 100–400 mg/day for primary hyperaldosteronism [48].

The remaining drugs further illustrate that formulation challenges are not limited to high-dose products. Carvedilol is typically titrated from 3.125 mg twice daily to 25 mg twice daily [49]; ezetimibe is administered at 10 mg once daily [50]; lurasidone is used at 20–160 mg once daily depending on indication [51]; and rivaroxaban regimens range from 2.5 mg twice daily to 20 mg once daily, with an initial 15 mg twice-daily regimen for acute deep-vein thrombosis or pulmonary embolism [52]. These labeled dose requirements demonstrate that poor aqueous solubility and dissolution-limited exposure can be relevant across low-, moderate-, and high-dose therapies. However, the present study demonstrates only the feasibility of producing nanoparticle-enriched formulations; any improvement in delivery or dose reduction must be established experimentally. Celecoxib and fenofibrate represent benchmark BCS Class II drugs where nanosizing and related formulation strategies have already been shown to improve dissolution and oral performance. A 2023 celecoxib nanoformulation study reported improved solubility, dissolution rate, and oral bioavailability, supporting the idea that nanoscale celecoxib formulations can create clinically relevant performance gains [8]. Additional celecoxib nanosuspension work supports the broader concept that particle-size reduction can enhance bioavailability [9]. Fenofibrate has a long history of formulation optimization, including micronized, nanoparticle, and other improved formulations; recent nanocrystal reviews also identify fenofibrate as a model compound for nanosizing and bioavailability enhancement [10,38,39]. FAST-generated versions of these benchmark drugs can therefore serve as internal positive comparators for determining whether FAST provides a simpler or faster route to similar formulation-enabling outcomes.

Itraconazole, rifampin, sorafenib, and spironolactone illustrate drugs for which FAST may support future route-specific or local-delivery reformulation. Itraconazole is a highly lipophilic azole with substantial formulation barriers, and prior nanoformulation studies support investigation of advanced delivery formats [14,15]. In the current study, representative TEM fields showed two itraconazole morphologies: rod-like electron-dense structures consistent with possible crystalline/nanocrystalline material and rounded nanoscale particles. The 0.22 μm filtration step enriched the filter-passing fraction analyzed by ZetaView/NTA, but fraction-specific composition and solid state were not established. Future studies should determine batch-to-batch reproducibility, recovery, drug content, solid-state properties, and release behavior before morphology-controlled reformulation is proposed, particularly for topical or local antifungal applications.

Rifampin nanoparticle and tuberculosis-delivery reviews emphasize the potential of nanoscale systems while also recognizing rifampin stability as a critical challenge [16,17]. In the current study, rifampin formed a measurable nanoscale particle population and showed no visible macroscopic sedimentation or precipitation. TEM revealed irregular clustered structures and rounded or oval particles, which may reflect rifampin self-association, concentration during grid drying, negative-stain/sample-preparation effects, or a combination of these factors. The aged suspension also showed visible darkening; however, visual inspection alone cannot establish whether chemical degradation occurred or identify degradation products. The color shift should therefore be treated only as a preliminary stability signal requiring future confirmation by HPLC or LC-MS.

Sorafenib drug delivery reviews highlight the importance of formulation strategies for overcoming poor solubility, low bioavailability, and dose-limiting toxicities [18]. Spironolactone nanoformulation reviews and topical delivery studies support the potential of nanoparticulate systems for targeted topical therapy in hyperandrogenic skin conditions and chronic wounds [19]. Carvedilol, ezetimibe, lurasidone, and rivaroxaban provide additional examples in which low solubility, food effects, solid-state behavior, or bioavailability limitations have motivated formulation strategies [11–13,20]. For each drug, FAST-generated nanoparticles should be viewed as rapid formulation prototypes that require comparison against existing approaches for drug loading, dissolution, stability, permeability, tissue targeting, and safety.

### Propofol: value-added reformulation of an approved hydrophobic anesthetic

Propofol was included as a challenging hydrophobic drug whose marketed formulation depends on an oil-in-water lipid emulsion. As shown in Figure 4, FAST processing generated spherical to near-spherical nanoscale particles by TEM, supporting the feasibility of preparing a nanoparticle-enriched aqueous propofol suspension. This proof-of-concept finding provides a basis for future comparison with the approved lipid-emulsion formulation; improved clinical performance cannot be inferred from the present data [21,22]. Approved propofol dosing is weight- and procedure-dependent. In healthy adults younger than 55 years, general-anesthesia induction commonly requires 2–2.5 mg/kg, while maintenance infusion rates are generally 100–200 micrograms/kg/min; lower doses or infusion rates are used for older, debilitated, or medically higher-risk patients and for sedation rather than general anesthesia [53]. These dosing requirements provide clinical context for the formulation challenge but do not imply that a FAST-generated propofol nanosuspension would achieve equivalent exposure or anesthetic performance.

Any potential formulation advantages remain hypothetical until drug content, free-drug concentration, release kinetics, sterility and endotoxin control, hemocompatibility, vascular irritation, cardiopulmonary safety, pharmacokinetics/pharmacodynamics, and anesthetic performance are established. Regulatory and alternative-route considerations are therefore conceptual. A 505(b)(2) pathway might be considered only if adequate CMC, nonclinical, and clinical bridging to an approved propofol product can be established [40]. Although the nasal route has been discussed broadly for central nervous system delivery [6,41,42], the present study provides no data supporting intranasal propofol; route-specific development should be reserved for future investigation.

### Significance of FAST-processed Omyadent HA100 hydroxyapatite nanoparticles

The preliminary Omyadent HA100 findings suggest that FAST may have broader applicability beyond hydrophobic organic drug molecules. Hydroxyapatite is an inorganic calcium phosphate material widely used in oral-care and dental applications because of its chemical similarity to mineral components of enamel and dentin [23–26]. Conventional commercial HA powders may be micron-sized or broadly distributed in particle size, which may limit access to nanoscale dental structures such as dentinal tubules, enamel surface defects, and microscopic mineral-loss regions. This provided the rationale for evaluating whether FAST processing could yield a nanoparticle-enriched suspension from a commercially relevant HA source under mild aqueous conditions. After FAST processing, Omyadent HA100 showed nanoscale HA clusters by NTA and TEM. The process did not involve conventional nanoparticle engineering approaches, including mechanical size reduction, high-pressure homogenization, surfactant stabilization, polymer encapsulation, or surface modification. The current data support the presence of nanoscale HA clusters after FAST processing, but without an untreated HA100 control they do not establish de novo nanoparticle formation or conversion of the starting material.

The NTA median particle size was 141.2 nm, with a measured range of 60.9–270.3 nm. Together with the TEM evidence of abundant irregular nanoscale HA clusters, these findings suggest that FAST processing produced a nanoparticle-enriched HA suspension. The broad size distribution and partial aggregation are not unexpected for mineral nanoparticles and may reflect both the intrinsic surface properties of HA and artifacts associated with drying during TEM preparation. Therefore, the current data should be interpreted as evidence of nanoscale HA clusters after FAST processing rather than complete conversion into a uniform population of isolated nanoparticles. From a dental-materials perspective, the formation of non-engineered nanoscale HA clusters may be important. Smaller HA particles may have improved access to microstructured tooth surfaces compared with larger micron-scale HA powders. Published oral-care literature supports the concept that nano-HA can deposit on tooth surfaces, contribute to remineralization processes, and occlude dentinal tubules in dentin hypersensitivity models [23–26]. In principle, FAST-processed nanoscale HA may better interact with enamel defects, dentin surfaces, and exposed tubules, although this functional advantage remains to be experimentally confirmed. Future studies should evaluate dentin tubule penetration, enamel surface deposition, remineralization potential, suspension stability, performance in relevant oral-care formulations, and cytocompatibility in oral keratinocytes, gingival fibroblasts, dental pulp or odontoblast-like cells, and inflammatory-response assays.

### Limitations and Next Steps

The present study establishes formulation feasibility rather than therapeutic performance. Future work should prioritize batch reproducibility, quantitative drug-content analysis, solid-state characterization, dissolution or release testing, and physical and chemical stability. Lead formulations would then require compound- and route-specific safety, pharmacokinetic, bioavailability, and efficacy studies. ZetaView/NTA and TEM provide complementary information, but filtration, drying, staining, aggregation, and sample-preparation conditions can influence apparent particle-size distributions and morphology. The number of independent formulation preparations and statistical variability for Table 2 should be reported when available, and the method used to define the measured size range should be specified.

## Conclusion

FAST generated aqueous nanoscale suspensions from a chemically diverse panel of ten BCS Class II drugs, propofol, and Omyadent HA100 hydroxyapatite. ZetaView/NTA analysis identified nanoscale particle populations with negative zeta potentials, visual assessment showed no macroscopic precipitation, sedimentation, aggregation, phase separation, or foam formation for the evaluated suspensions, and TEM imaging supported diverse nanoscale morphologies for HA100, propofol, itraconazole, and rifampin. Itraconazole showed coexisting rod-like electron-dense structures consistent with possible crystalline/nanocrystalline material and rounded nanoscale particles, supporting future investigation of morphology-controlled reformulation, particularly for topical or local delivery. Rifampin showed heterogeneous rounded/oval particles and irregular clustered structures by TEM and a clear aging-related color shift, highlighting the need for drug-specific physical and chemical stability testing. For HA100, the findings support the presence of nanoscale clusters after FAST processing but do not establish de novo nanoparticle formation from the starting material. The findings support FAST as a rapid carrier-free formulation-feasibility platform, but additional studies are required to establish dissolution enhancement, chemical stability, drug loading, safety, pharmacokinetics, route-specific delivery, and efficacy. If confirmed in future studies, FAST may provide a practical platform for early formulation screening, AI-assisted drug discovery, value-added reformulation of approved drugs, and inorganic oral-care material development.

## Figures and Tables

**Figure 1:**
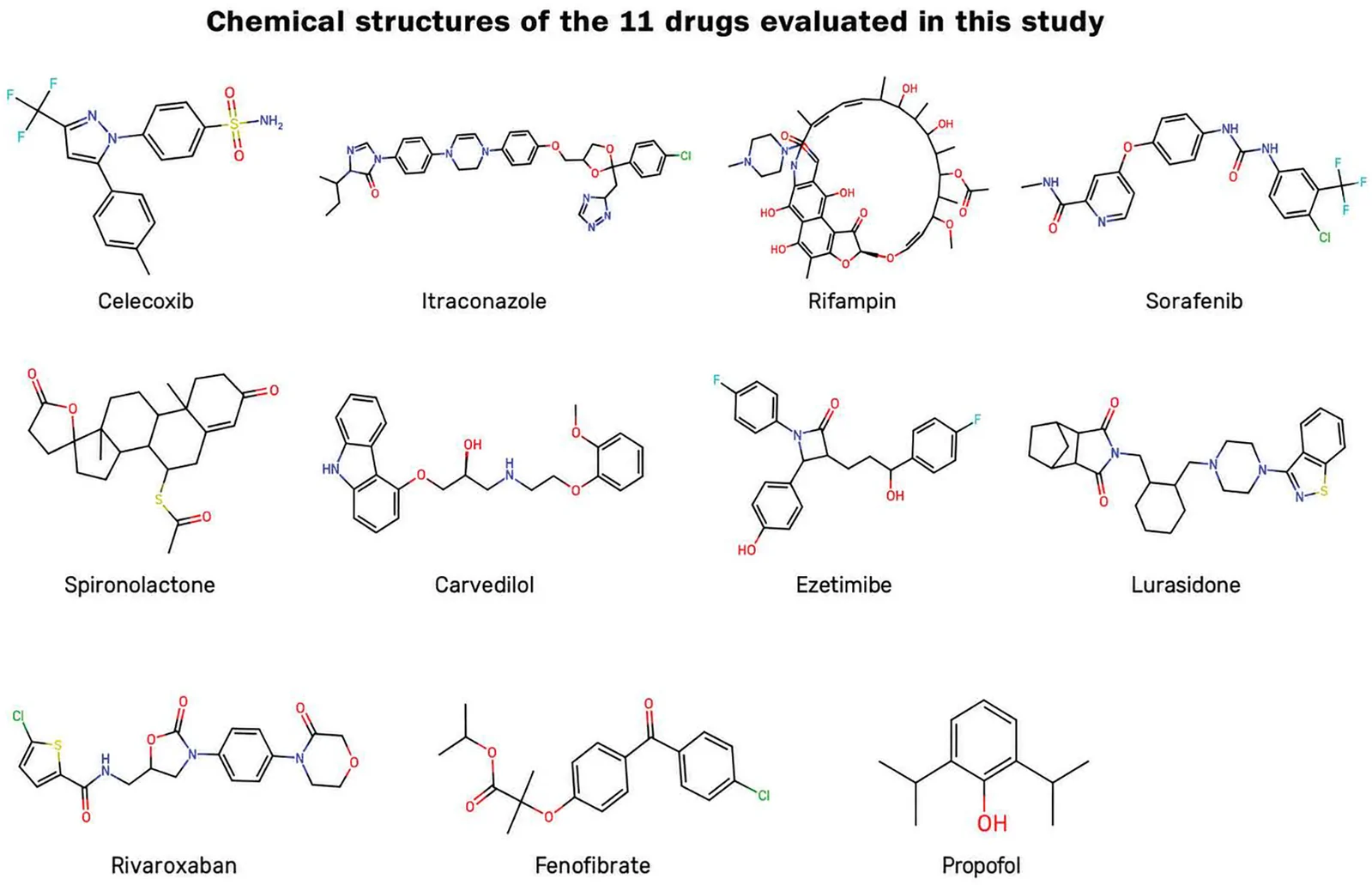
Structural and physicochemical features of the selected drugs Chemical structures of the 11 drugs evaluated in this study. The figure includes celecoxib, itraconazole, rifampin, sorafenib, spironolactone, carvedilol, ezetimibe, lurasidone, rivaroxaban, fenofibrate, and propofol.

**Figure 2:**
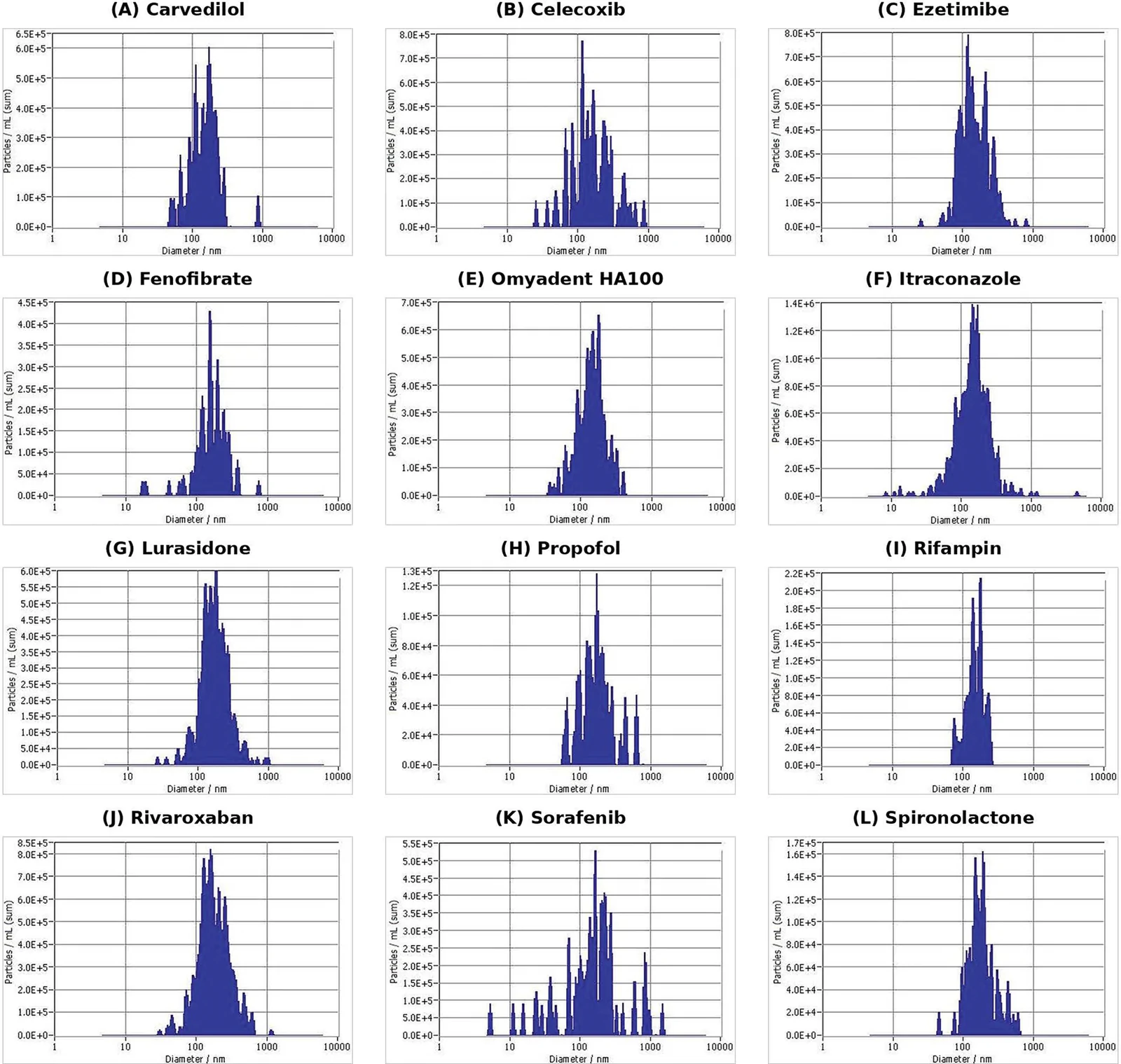
Particle-size distribution profiles of FAST-generated aqueous nanoscale suspensions. (A) Carvedilol, (B) Celecoxib, (C) Ezetimibe, (D) Fenofibrate, (E) Omyadent HA100, (F) Itraconazole, (G) Lurasidone, (H) Propofol, (I) Rifampin, (J) Rivaroxaban, (K) Sorafenib, and (L) Spironolactone. Profiles were obtained using ZetaView/NTA particle-size distribution analysis after dilution in water. Omyadent HA100 was included as an inorganic hydroxyapatite proof-of-concept material and is not a BCS Class II small-molecule drug. Note that the y-axis scale differs by panel.

**Figure 3:**
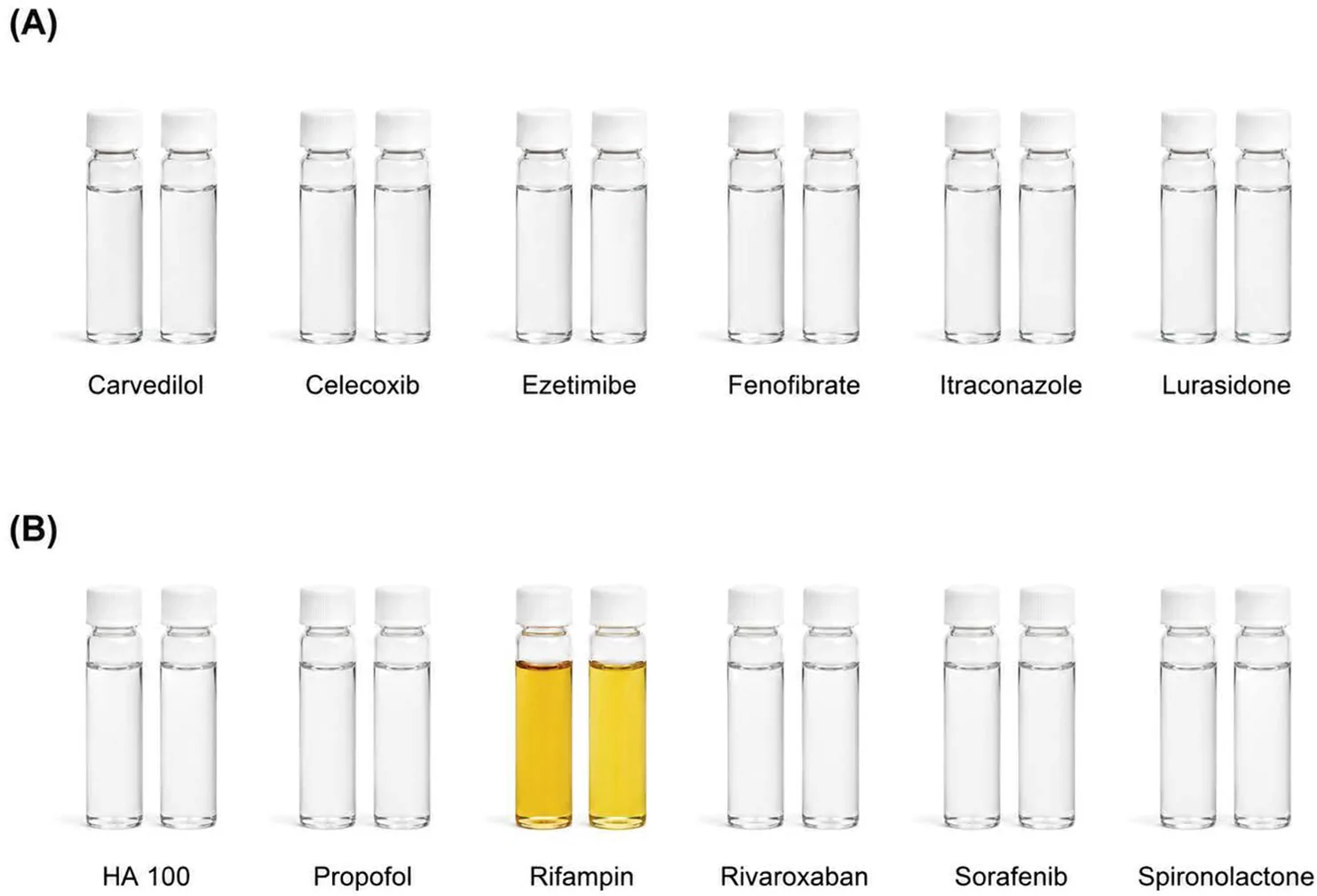
Visual appearance and short-term stability assessment of FAST-generated aqueous suspensions. Each pair shows aged (left) and freshly prepared (right) suspensions for the indicated material. (A) Group 1: Carvedilol, Celecoxib, Ezetimibe, Fenofibrate, Itraconazole, and Lurasidone. (B) Group 2: HA100, Propofol, Rifampin, Rivaroxaban, Sorafenib, and Spironolactone. No visible precipitation, sedimentation, aggregation, phase separation, or foam formation was observed by visual inspection. Rifampin showed the most apparent color change, with the aged suspension appearing darker yellow/amber relative to the freshly prepared suspension.

**Figure 4:**
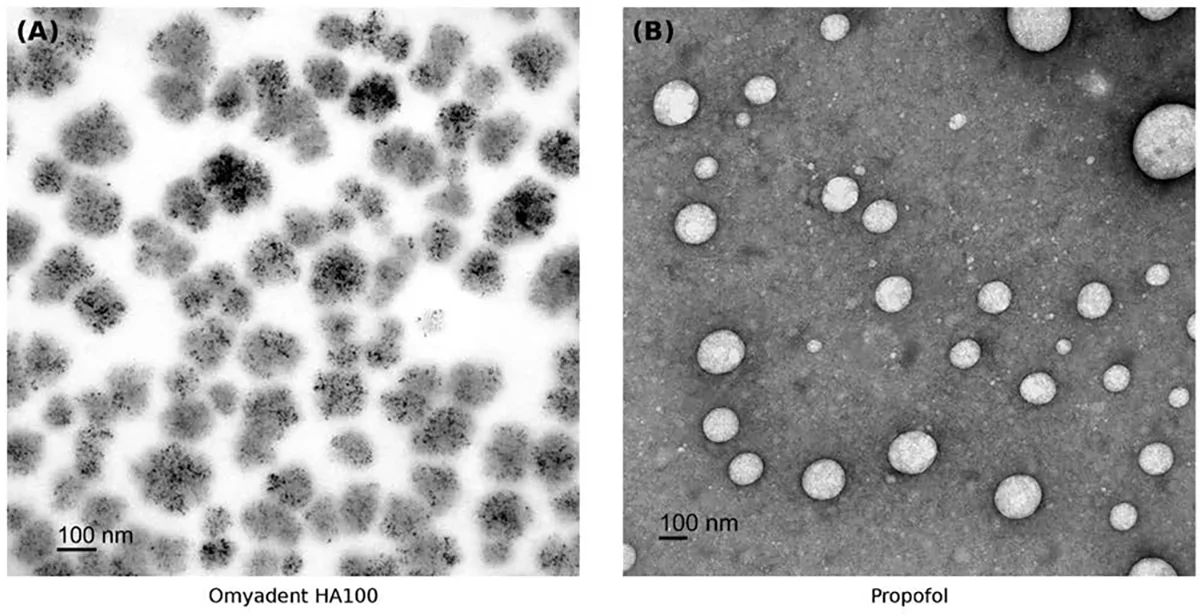
Transmission electron microscopy of selected FAST-generated nanoparticles. (A) Omyadent HA100 hydroxyapatite showed electron-dense, irregular granular nanoscale clusters distributed across the examined field. Some larger aggregates were also observed, and no obvious long needle-shaped HA particles were observed. (B) Propofol showed discrete spherical to near-spherical nanoparticles with nanoscale morphology and some size heterogeneity. Scale bars = 100 nm.

**Figure 5:**
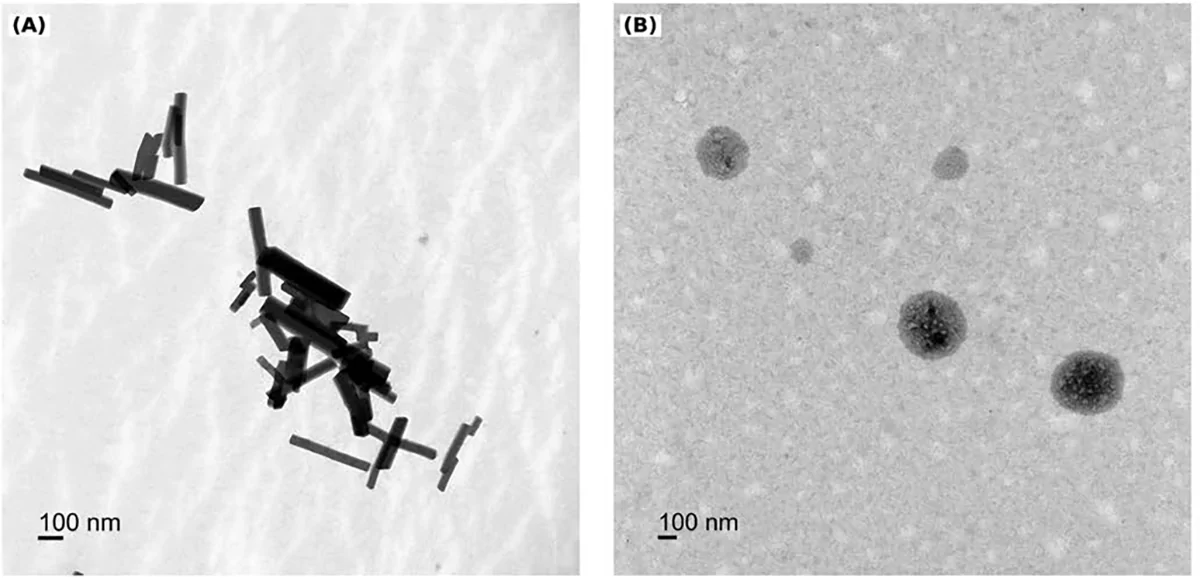
Transmission electron microscopy of FAST-generated itraconazole. (A) Rod- or needle-like electron-dense nanoscale/submicron particles consistent with possible crystalline/nanocrystalline itraconazole structures from unfiltered stock preparation. (B) Rounded itraconazole nanoscale particles were also observed, indicating mixed morphology within the FAST preparation. Scale bars = 100 nm.

**Figure 6:**
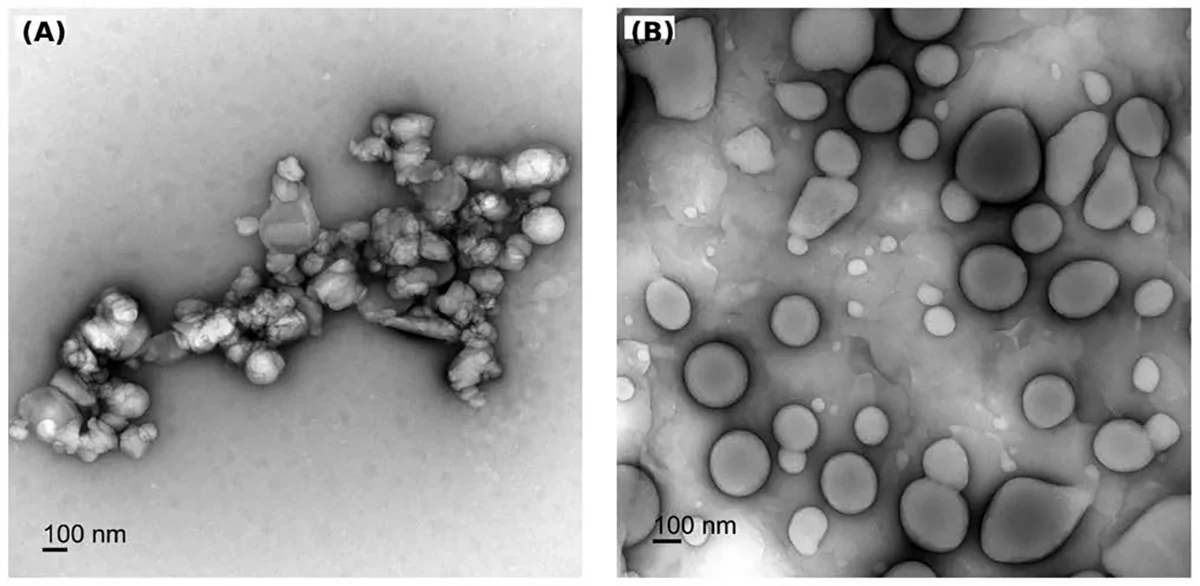
Transmission electron microscopy of FAST-generated rifampin. (A) Representative rifampin field showing irregular aggregated clusters composed of smaller nanoscale substructures. (B) Representative field showing rounded to oval nanoscale particles within a diffuse matrix. Scale bars = 100 nm.

**Table 1:** BCS Class II drugs, propofol, and hydroxyapatite evaluated and rationale for selection.

Material	Category / use context	Formulation-relevant rationale	FAST evaluation purpose
Celecoxib	Anti-inflammatory drug	Benchmark poorly soluble oral drug; nanosizing has been used to improve dissolution and exposure [8,9,27].	Test FAST against a well-studied BCS Class II benchmark.
Fenofibrate	Lipid-lowering drug	Classic poorly soluble drug with a long history of formulation optimization [10,36].	Benchmark FAST against a highly lipophilic oral drug.
Itraconazole	Azole antifungal drug	Highly lipophilic drug with topical, ocular, local, and systemic formulation barriers [14,15,28].	Evaluate the filter-passing nanosuspension fraction and coexisting rod-like and rounded morphologies.
Rifampin	Rifamycin antibiotic	Key tuberculosis drug with aqueous formulation and stability challenges [16,17,29].	Assess nanoscale dispersion and stability-sensitive morphology.
Sorafenib	Anticancer multikinase inhibitor	Poor aqueous solubility and limited bioavailability motivate delivery-system development [18,30].	Assess FAST feasibility for an oncology-relevant hydrophobic drug.
Spironolactone	Steroid-like dermatology/cardiovascular drug	Hydrophobic scaffold; topical nanoformulations have been investigated for skin disorders [19,31].	Assess FAST feasibility for topical/local reformulation concepts.
Carvedilol	Cardiovascular beta-blocker	Low solubility and poor bioavailability are major formulation barriers [12,32].	Assess FAST feasibility for an amphipathic oral drug.
Ezetimibe	Lipid-lowering drug	Poor dissolution has motivated multiple formulation strategies [11,33].	Assess FAST feasibility for an oral lipid-lowering drug.
Lurasidone	CNS-active drug	Poor aqueous solubility and food effect create formulation limitations [20,34].	Assess FAST feasibility for a CNS-relevant poorly soluble drug.
Rivaroxaban	Oral anticoagulant	Poor solubility can reflect solid-state behavior and molecular packing as well as lipophilicity [13,35].	Assess FAST feasibility for a clinically important oral drug.
Propofol	Hydrophobic intravenous anesthetic	Approved product depends on an oil-in-water lipid emulsion [21,22,37].	Stress-test FAST for value-added reformulation of an approved hydrophobic drug.
Omyadent HA100	Inorganic hydroxyapatite oral-care material	Hydroxyapatite and nano-HA are relevant to remineralization and dentin hypersensitivity research [23–26].	Test whether FAST processing yields a nanoparticle-enriched suspension from an inorganic dental material.

**Table 2:** Summary of FAST-generated aqueous nanoscale suspensions measured by ZetaView/NTA and zeta potential analysis. Unless otherwise indicated, values represent aqueous nanoparticle suspensions prepared by diluting FAST stock suspensions to a nominal 0.02% working suspension level before analysis. The percentage value is a preparation/dilution designation and should not be interpreted as an analytically confirmed w/v or w/w drug concentration.

Material	Nominal working suspension level	Median size (nm)	Measured size range (nm)	Particle concentration (particles/mL)	Zeta potential (mV)
Carvedilol	0.02%	145.5	91.3–172.5	6.6 × 10^8	−46.93
Celecoxib	0.02%	149.5	32.0–242.8	4.51 × 10^8	−35.72
Ezetimibe	0.02%	137.3	92.3–276.0	1.9 × 10^9	−44.53
Fenofibrate	0.02%	157.0	119.3–282.3	3.5 × 10^8	−28.21
Itraconazole*	0.02%	150.8	67.0–236.0	1.8 × 10^9	−41.1
Lurasidone	0.02%	168.0	74.7–220.1	8.1 × 10^8	−54.94
Propofol	0.02%	150.47	95.3–202.5	5.3 × 10^8	−38.12
Rifampin	0.02%	145.5	75.4–225.4	3.1 × 10^8	−37.45
Rivaroxaban	0.02%	165.5	128.9–254.7	2.4 × 10^9	−42.36
Sorafenib	0.02%	159.2	100.9–269.3	1.4 × 10^9	−29.9
Spironolactone	0.02%	145.5	67.3–172.5	6.6 × 10^8	−51.15
Omyadent HA100	0.02%	141.2	60.9–270.3	7.8 × 10^8	−22.07

* Itraconazole values represent the 0.22 μm filter-passing nanosuspension fraction after removal of larger rod-like crystalline/nanocrystalline structures.
